# Comprehensive Insights into Monkeypox (mpox): Recent Advances in Epidemiology, Diagnostic Approaches and Therapeutic Strategies

**DOI:** 10.3390/pathogens14010001

**Published:** 2024-12-26

**Authors:** Suresh Kumar, Dhanyashri Guruparan, Kalimuthu Karuppanan, K. J. Senthil Kumar

**Affiliations:** 1 Faculty of Health and Life Sciences, Management and Science University, Shah Alam 40100, Malaysia; sureshkumar@msu.edu.my (S.K.); dhanyag@gmail.com (D.G.); 2 Department of Biotechnology, School of Bioengineering, SRM Institute of Science and Technology, Kattankulathur, Chennai 603203, Tamil Nadu, India; kalimutk1@srmist.edu.in; 3 Center for General Education, National Chung Hsing University, Taichung 402, Taiwan

**Keywords:** orthopoxvirus, monkeypox, zoonotic diseases, vaccine

## Abstract

Monkeypox (mpox) is a viral infection closely related to smallpox, manifesting as a milder febrile rash in affected individuals. Over the past two decades, the incidence of mpox has surged, possibly linked to a declining immunity against the smallpox vaccine worldwide. Recent outbreaks of mpox in multiple countries have sparked concerns regarding altered transmission patterns and the potential for a global menace. In this article, we present a multidimensional review encompassing the latest scientific discoveries, illuminating the intricate structure of the human mpox virus. Key findings include advancements in understanding the virus’s molecular mechanisms, which highlight its genetic adaptability and potential for zoonotic spillover. Diagnostic innovations, such as improved molecular assays, have enhanced detection accuracy, while novel therapeutic strategies, including antiviral drugs and vaccines, show promise in mitigating outbreaks. Our conclusions emphasize the importance of robust surveillance systems, vaccination programs, and rapid response strategies to curb mpox’s spread. Future recommendations include strengthening global collaboration for zoonotic disease surveillance, advancing the research on host–pathogen interactions, and developing next-generation therapeutics to address this emerging public health threat effectively.

## 1. Introduction

Monkeypox (mpox) is a viral disease and an agent of the genus Orthopoxvirus. The illness, which is primarily seen in Central and West Africa, is comparable to smallpox, which was declared extinct in 1980. As a zoonotic virus, the mpox virus most frequently infects humans through contact with rodents and primates that have the disease. Fever, chilliness, enlarged lymph nodes, headache, back pain, muscle aches, and fatigue are some of the symptoms of mpox. A rash typically starts on the face before spreading to other body parts. mpox can occasionally result in serious disease and even death, especially in people with weakened immune systems. The World Health Organization (WHO) has recommended adding mpox to its list of conditions that could cause a public health emergency of global concern [1]. The disease was identified in humans for the first time in 1970, and periodic outbreaks have been noted. It is common in certain parts of Central and West Africa. Uncertainty still exists over the extent of mpox transmission in Africa, which may be underreported [2]. In recent years, few cases of mpox have been documented in the United States and the United Kingdom, most usually among travelers from the affected African nations. Given that the mpox virus is a zoonotic disease, it is crucial that we understand the role that wildlife plays in its maintenance and dissemination. Hence, to control and halt the spread of the illness, a study on the epidemiology, ecology, and monitoring of mpox is essential [3,4,5]. This review aimed to provide a comprehensive understanding of monkeypox by integrating recent advancements in molecular characterization, epidemiology, clinical manifestations, diagnostic tools, and therapeutic strategies. It aims to offer valuable insights for virology research and public health measures to combat this emerging global health threat.

## 2. Background and Issues

mpox was initially identified in research laboratory monkeys in 1958 [6]. The virus is frequently found in monkeys and rodents in Central and West Africa, where mpox has proven to be particularly dangerous in children, with death rates as high as 10% in severe outbreaks [7]. Additionally, infected ”exotic pets” such as enormous, pouched rats, brush-tailed porcupines, and rope squirrels have spread the mpox virus outside Africa. The first mpox pandemic outside Africa occurred in the United States of America in 2003, when a caged prairie dog in Milwaukee, Wisconsin transmitted the virus to a child [8]. There were a few rare cases recorded in the UK, US, and Singapore between 2018 and 2022; in each of them, the underlying index case was a person who had recently left Nigeria [9,10,11,12]. 

The mpox virus can infect humans by animal bites or via direct contact with contaminated human bodily fluids. It can also be spread from person to person by intimate contact, most typically amongst family members; exposure to respiratory droplets may also be a mechanism of transmission [13]. The illness reveals itself in people two weeks after infection, with fever, headache, general malaise and tiredness, and swollen lymph nodes. A few days later, a rash of elevated pimples appears on the face and torso. They eventually crust and fall off, and the illness takes two to four weeks to run its course. Outbreaks are kept under control by isolating patients and keeping the environment clean [14]. To prevent infection, Jynneos (Imvanex or Imvamune), an attenuated live virus vaccine, can be utilized [15,16]. Smallpox vaccination similarly provides some defense against the mpox virus; smallpox vaccine injection may help prevent persons who are at risk of being infected with the mpox virus, such as veterinarians and other animal workers [17]. 

A more serious outbreak that included widespread human-to-human spread outside of Africa started in 2022. In May 2022, the outbreak started in the UK and spread quickly in the months that followed, infecting countries in Africa, Asia, Australia, and the Americas. Traditionally, mpox was considered a zoonotic disease. Nevertheless, many of the reported cases in recent outbreaks have been linked to intimate and sexual contact, particularly among men who have sex with men (MSM). This pattern has raised concerns about mpox’s association with sexual health [18]. Although mpox is not classified as a sexually transmitted infection (STI), evidence suggests that the virus can be present in bodily fluids, including semen, and transmission during close physical contact is likely. The clustering of cases within sexual networks has highlighted the need for targeted awareness campaigns and interventions within these communities. The sexual health aspect of mpox underscores the importance of reducing stigma, promoting accurate health messaging, and integrating mpox prevention efforts into existing sexual health services. Addressing these challenges is crucial to controlling the outbreak and preventing future spread [19]. The WHO declared the monkeypox outbreak a Public Health Emergency of International Concern in late July, after more than 18,600 cases were reported [20,21,22,23,24]. In 1958, human mpox, which is connected to the smallpox virus, was discovered [25]. The present epidemic in 2022 has been puzzling because there is no epidemiological link and there is a risk that the virus might be spread sexually. In addition, the morphological and pathogenetic mechanisms by which the virus enters host cells are poorly understood. However, a recent national multicenter study (SEIMC-CEME-22) in Spain analyzed the early phase of the 2022 mpox outbreak. It included 1472 confirmed cases, primarily affecting middle-aged cisgender men (99%), with sexual exposure, especially among men who have sex with men (MSM), as the main transmission route. Immunosuppression, mostly due to HIV, was observed in 40% of patients. Common symptoms included rash (95.7%), fever (48.2%), and adenopathies (44.4%). Co-infections like syphilis, gonorrhea, and HIV was frequent. Only 6.5% had prior orthopoxvirus vaccination. Treatments were heterogeneous, with most receiving symptomatic care; 58 patients required hospitalization, and 1 died. This highlights the need for targeted public health interventions [26,27]. This article seeks to summarize what is now known about this re-emerging virus and speed up the understanding of previously published research findings on the human mpox virus.

## 3. mpox Virus Classification

The Orthopoxvirus (OPXV) genus of the Poxviridae family contains the mpox virus, Poxviruses are enclosed big viruses; 200 kilobase pairs of double-stranded DNA (dsDNA) that are heavily loaded with 200 genes make up their genome [28,29]. Around half of the genes are well-evolved in vertebrate poxviruses and are required for viral replication, while the other half are referred to as “accessory” genes that are mostly involved in virus and host interactions and may not be necessary for viral replication independently [30]. Many poxviruses infect most invertebrates and vertebrates, affecting a wide range of veterinary and human disorders. The two subfamilies that make up the Poxviridae family are Entomopoxvirinae, which infects insects, and Chordopoxvirinae, which infects vertebrates. One of the 11 genera that make up the subfamily Chordopoxvirinae is OPV (Table 1). All OPV species are categorized as zoonotic viruses since they all have animal reservoirs except the variola virus, which only affects humans [31,32].

## 4. mpox Genetic Clades and Evolution

Experts in virology, evolutionary biology, and councils of research institutes from around the world reviewed the phylogeny and nomenclature of known and unknown mpox virus variations or clades. They discussed the characteristics and evolution of several mpox virus strains, differences in their apparent phylogenetic and clinical composition, and any potential repercussions for virological and evolutionary research, as well as public health in the future. The panel decided on new nomenclature for the virus clades that adheres to professional norms. They reached an agreement on the listing and categorization of virus clades on genomic sequence repository sites [33,34,35].

In the proper naming system, the clade will be denoted by a Roman number, and the subclades will be denoted by a lower-case alphanumeric character. Hence, Clade I, Clade IIa, and Clade IIb are included in the revised terminology, with Clade IIb denoting the collection of variants that were primarily in use during the global pandemic of 2022 [5]. Lineages will be given the names that the experts have suggested as the pandemic progresses. If further time is required, the experts will convene. The new names for the clades should go into force immediately, while work on the names of the diseases and viruses continues [36,37]. Different strains of the monkeypox virus (MPXV) exhibit genetic polymorphism and genomic instability, contributing to strain evolution. MPXV is a DNA virus that undergoes frequent mutations, enabling its rapid spread. The emergence of well-adapted variants can have devastating global consequences. Minor genetic changes facilitate host adaptation, with moderate transmission rates being more common. Genetic mutations, both stabilizing and destabilizing, enhance viral fitness and support interhuman transmission. Different strains of MPXV exhibit variations in their genome sizes, ranging from 190,083 to 206,372 base pairs. These strains are classified into two clades: West African and Congo Basin. Through epidemiological studies, researchers have observed differences in the severity of MPX infection in various regions [38]. By comparing three West African strains (SL-V70, complement control protein [COP]-58, and WRAIR-61) with a Central African strain (ZAI-96), it was found that there is a nucleotide variance of approximately 0.55% to 0.56%. The primary distinctions between these strains lie in the orthologs of the *BR-203C*, *BR-209*, and *COP-C3L* genes. The BR-203 gene, for instance, encodes a complete protein comprising 221 amino acids [39]. Curiously, the West African strain of MPXV encodes only a partial *N*-terminal fragment, consisting of approximately 51 amino acids, for the specific gene under investigation. Intriguingly, this gene is an ortholog of the M-T4 gene that exists in the myxoma virus [39]. The myxoma virus is notorious for causing myxomatosis, a disease affecting European rabbits. Intriguingly, scientific studies have shown that deleting the M-T4 gene results in a heightened inflammatory response. This finding suggests that this gene regulates the immune response to viral infections. 

BR-209 acts as an IL-1β-binding protein, preventing the binding of IL-1β to the IL-1 receptor. The cytokine IL-1, present in forms of the IL-1α, IL-1β, and IL-1 receptor antagonist, affects the inflammatory response. One of these mechanisms involves the production of vIL-1βBP. By binding to IL-1β, vIL-1βBP prevents IL-1β from interacting with its cellular receptors, inhibiting its signaling and dampening the immune response [40]. The *COP-C3L* gene in the vaccinia virus codes for a secreted protein called vaccinia virus COP (VCP). The Central African strain of MPXV expresses a shorter protein known as the MPX inhibitor of complement enzymes (MOPICE), which is the ortholog of the *COP-C3L* gene. The variola virus, responsible for smallpox, contains the ortholog known as the smallpox inhibitor of complement enzymes (SPICE). MPXV strains exhibit genetic diversity, with the Congo Basin clades being more severe and associated with human infections worldwide [41]. Understanding the roles of these genes in MPXV pathogenesis is essential for comprehending the complex interactions and their contributions to the virus’s disease-causing mechanisms. The 2022 outbreak of MPXV is associated with a divergent branch, referred to as lineage B.1, which is derived from lineage A.1. Lineage A.1 has been associated with MPXV exports from Nigeria to the United Kingdom, Israel, and Singapore in 2018–2019 [35].

The 2022 MPXV outbreak may be attributed to the ongoing spread and evolution of the virus from the 2017–2018 Nigeria outbreak [42]. Poxviruses, including MPXV, have a lower mutation rate compared to RNA viruses. However, the 2022 MPXV shows a rapid divergence from the 2018 virus, suggesting a rapid adaptation to its host. APOBEC3 editing contributes to the mutation rate, with 90% of new nucleotide changes due to this process. Recombination plays a crucial role in poxvirus evolution, with the first natural recombination event in MPXV [43]. Gene loss and amplification are important factors in MPXV’s evolution, with the West African clade having larger genomes and more gene content. Monitoring non-synonymous mutations, genome ends, and gene content is crucial for understanding the evolutionary dynamics and possible adaptations of MPXV. The mpox virus’s development and transmission are influenced by point mutations in several proteins, leading to the multi-country outbreak in 2022. The virus originated from the MPXV/United States/2021/MD virus and has evolved through ten common amino acid alterations. The B.1 viruses, which were transferred from Nigeria between 2018 and 2019, have specific amino acid changes in 22 of the 26 proteins. The study also examined characteristics of codon use and host adaptability, revealing a bias in codon use in genes undergoing nucleotide alterations in the B.1 lineage. Selection pressure, rather than mutation pressure, played a significant role in the evolution of genes expressing nucleotide mutations in the B.1 lineage. Monitoring and analyzing the virus’s genetic alterations is crucial for improving our understanding of its behavior and guiding effective preventative and control efforts [33].

## 5. mpox Epidemiology

Sub-Saharan Africa has possibly seen mpox for several thousands of years since humans initially contracted the virus via close contact with diseased animals [44]. mpox was recognized as a distinct sickness after smallpox was eradicated in 1970 and it became clear that smallpox-like infections were still happening in rural areas [44]. The mpox virus was first discovered in monkeys used for research in 1958 at State Serum Institutes in Copenhagen, Denmark, and Africa. mpox has received attention as a disease of significant global public health concern since the outbreak of the first epidemic in the USA in 2003, which was linked with infected pet prairie dogs [45]. It was assumed that the native prairie dogs housed with imported rodents from Ghana in the western region of Africa were the primary source of the outbreak because the bulk of the affected people became ill after interacting with pet prairie dogs. mpox has been connected to a rise in cases that began in the Midwest of the US in the summer of 2003. mpox cases have been reported in several countries since 2003, with the worst epidemic occurring in Nigeria in 2017 [45].

**Figure 1:**
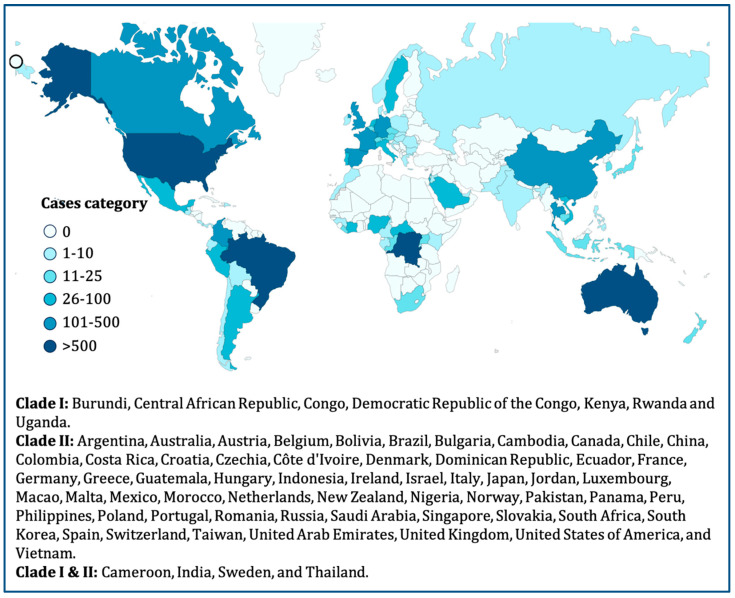
Global map of mpox outbreak in 2024 from CDC (1 January 2024).

The Centres for Disease Control and Prevention (CDC) had reported 5783 instances of mpox as of 1 July 2022, spread over 52 different nations. Figure 1 shows the geographical distribution of the cases worldwide. Currently, the western hemisphere and parts of Europe are home to the majority of mpox cases. According to recent studies, the United Kingdom has the most instances in all of Europe. With a median age of 31 years, most cases reported of mpox currently involve people under the age of 40. This group only emerged after the smallpox vaccination campaign had been abandoned, further illuminating the lack of cross-protective immunity [14]. According to the External Situation Report issued on 10 June 2023, the World Health Organization (WHO) received reports of 87,929 laboratory-confirmed cases of mpox from 111 countries across all six WHO Regions between 1 January 2022, and 5 June 2023. This totals 146 deaths. Since the last situation update, 552 new cases (a 0.6% rise) and 6 new fatalities have been reported. The number of new cases worldwide fell somewhat in the most recent reporting week. The Americas have recorded the most cases in the last three weeks. Cases have increased in several countries, including Cameroon. Europe and South-East Asia have also reported additional cases, but with some delay [46,47]. The number of cases in Africa has dramatically grown, exceeding the previously recorded average. As of 5 June 2023, 15 nations had confirmed new cases within the maximum illness incubation time. The United States, Brazil, and Spain are among the countries with the most cumulative instances. These 10 countries account for 83.9% of all instances globally. Since 1 January 2024, 13 African countries have reported mpox outbreaks, with a total of 18,737 cases, including 3101 confirmed and 16,636 suspected cases. The outbreak has resulted in 541 deaths, leading to a case fatality ratio of 2.89% [46,47]. The affected countries include Burundi, Cameroon, the Central African Republic, the Republic of the Congo, Côte d’Ivoire, the Democratic Republic of the Congo (DRC), Ghana, Kenya, Liberia, Nigeria, Rwanda, South Africa, and Uganda [48]. On 15 August 2024, Sweden confirmed its first case of mpox Clade I, with the infection traced to a region in Africa experiencing a significant outbreak of this variant [48].

This case marked the first recorded instance of Clade I mpox outside of Africa. As of 16 August 2024, the Democratic Republic of the Congo (DRC) has reported a total of 16,794 mpox cases, including 2860 confirmed and 14,934 suspected cases, with 535 fatalities, resulting in a case fatality ratio of 3.19%. The outbreak has spread across all 26 provinces, predominantly affecting males and children under 15 years of age. The persistence of widespread transmission emphasizes the need for targeted interventions, particularly in vulnerable groups and high-risk regions. Burundi officially declared a mpox outbreak on 25 July 2024 [49]. By 16 August 2024, the country had reported 399 cases, comprising 100 confirmed and 299 suspected cases, with no fatalities. The mpox Clade IB variant was identified in confirmed cases. Among those affected, 55% were males, and children under five years old made up 38% of the confirmed cases. The outbreak has spread to 23 out of the 49 districts, highlighting the need for focused public health measures and surveillance. By 16 August 2024, a total of 263 cases had been reported in The Central African Republic (CAR), including 40 confirmed and 223 suspected cases, with no deaths. The outbreak affected six out of the country’s seven health regions, with 62% of cases occurring in males. Additionally, a significant proportion of cases (43%) were among children under five years of age, indicating the virus’s impact on vulnerable populations [50]. Rwanda declared its first mpox outbreak on 27 July 2024. As of 19 August 2024, the country had reported four confirmed cases with no associated deaths. This marks the first occurrence of mpox transmission in Rwanda, prompting health authorities to implement response measures to contain the spread and prevent further cases [47]. As of 16 August 2024, Nigeria’s Ministry of Health reported nine confirmed mpox cases with no deaths. The outbreak affected six of the country’s seven health regions, with the majority of cases (70%) occurring in males. Additionally, 38% of the cases involved children under the age of 10, indicating a significant impact on young populations [48].

The monkeypox outbreak continues, with low levels of transmission in Europe and the Americas, a minor decline in the Western Pacific, and an increase in cases in South-East Asia. The number of cases has recently increased in Africa, where transmission is more constant. Most patients are men, and the age distribution is consistent. Children make up a minor fraction of cases, with the majority being recorded in the Americas Region [47]. Transmission occurs largely among homosexual, bisexual, and other males who have intercourse with other men. The predominant mechanism of transmission is through skin and mucosal contact during sex, followed by non-sexual contact between people. The most typically reported exposure contexts are parties involving sexual interaction; however, this is changing with time [51]. Rash, fever, systemic rash, and vaginal rash are all common symptoms [52]. However, reliable information on transmission and exposure settings is scarce in the WHO African Region, making it difficult to gain thorough knowledge of the virus’s spread there.

## 6. Viral Re-Emergence of mpox

On 18 May 2022, Portugal, Spain, and Canada reported 14, 7, and 13 cases of MPV infection, respectively. On 19 May 2022, Belgium, Sweden, and Italy confirmed their first MPV instances. On 20 May, Australia reported two incidents. On 20 May 2022, France, and Germany, as well as the Netherlands all reported their first cases. On 20 May, the UK’s Health Secretary revealed an additional 11 MPV cases, bringing the total to 71. The first nation to impose a 21-day MPV quarantine requirement was Belgium. On 21 May 2022, Israel and Switzerland both verified their initial instances [53]. On 18 May 2022, Spain confirmed the very first case. The Republic of Spain reportedly reported a spike in cases, at 20, on 3 June, bringing the overall number of cases to 186. Denmark’s first incidence was reported on 23 May. This was said to be a visitor who had just arrived from the Canary Islands. On 24 May 2022, Quebec in Canada reported the confirmation of 15 instances; the same day, the Czech Republic also announced the confirmation of one case. In Belgium, the putative offender attended a concert event. The country’s first confirmed case was a 29-year-old tourist from West Africa who visited the United Arab Emirates at the end of May. Slovenia also verified its initial incident [54].

We found that 73 nations reported MPV cases as of 1 January 2024. The origin of the present MPV outbreak, nevertheless, is yet unknown. According to the MPV’s changing nature, it may be transmitted from person to person or from animal to person. Travelers from the endemic areas of Africa to North America and Europe were the ones who initially contracted the illnesses, which subsequently spread (Figure 1) [55]. The resurgence of mpox has led to debate, with declining immunity being a major theory. The theory is that waning immunity from previous smallpox vaccinations, which conferred some cross-immunity to MPXV, contributed to the rise in mpox cases. In the 1980s, the cessation of widespread smallpox vaccination made the population more susceptible to mpox, leading to selection pressure on the virus. This selection pressure may have triggered the development of mechanisms to bypass the immune system in MPXV, increasing the transmissibility of the virus. A second theory suggests that nonsynonymous mutations, particularly in coding regions associated with host recognition elements, may facilitate virus adaptation and fitness. These mutations could help the virus evade host immune responses and increase its transmission potential. Because of its affiliation with this phylogenetic branch, the emergence of specific mutations in the genomes of lineage B.1 MPXV compared with related viruses has attracted considerable attention in 2018–2019. These mutations suggest accelerated microevolutionary processes that could lead to an increase in human-to-human transmission. The B.1 MPXV lineage responsible for the current pandemic on the European continent probably first appeared in March 2022. This finding suggests that the B.1 lineage evolved and spread in Europe, resulting in the first MPXV cases that later spread to other continents, such as Oceania and the Americas. Recent analyses also indicate that MPXV has adapted more rapidly in the last two years, suggesting an accelerated process of host adaptation. These results provide evidence for the adaptability of MPXV and its dynamics in human populations. The proposed theories offer possible explanations for the resurgence of mpox cases and highlight the importance of monitoring and understanding the evolution of the virus and transmission patterns to support public health.

## 7. mpox Virus Transmission

The mpox virus spreads to and between humans when a person comes into proximity to an infected animal, individual, or objects that are contaminated. In addition to moving in large airborne droplets that invade the body via the mouth, eyes, or nose, the virus can also enter via damaged skin (even undetectable wounds). For efficient human-to-human transmission, continuous contact is essential since these droplets are dense and seldom move more than a few feet. As an outcome, healthcare personnel and those who live in the same household as an infected person are more vulnerable [13,56].

Among the 2022 outbreak, mpox cases have indeed been concentrated in males who have had sex with men (MSM). Although infectious MPXV has been found in sperm, MPXV DNA has recently been detected in urine, feces, rectal swab, nasopharyngeal swab, and saliva. However, it is unknown whether the virus may infect sperm cells and reproduce in the genital canal. It is still unknown and under inquiry whether mpox may be spread especially via sexual transmission methods. In any case, many are speculating if the virus has mutated to make easier human-to-human transmission possible, similar to SARS-CoV-2, in light of this pattern of transmission and the emergence of mpox in non-endemic locations. mpox is less likely to inherit changes that speed up its spread since it is a DNA virus, which can detect and correct mutations effectively than RNA viruses such as SARS-CoV-2 [57].

## 8. mpox Virus Morphology

**Figure 2:**
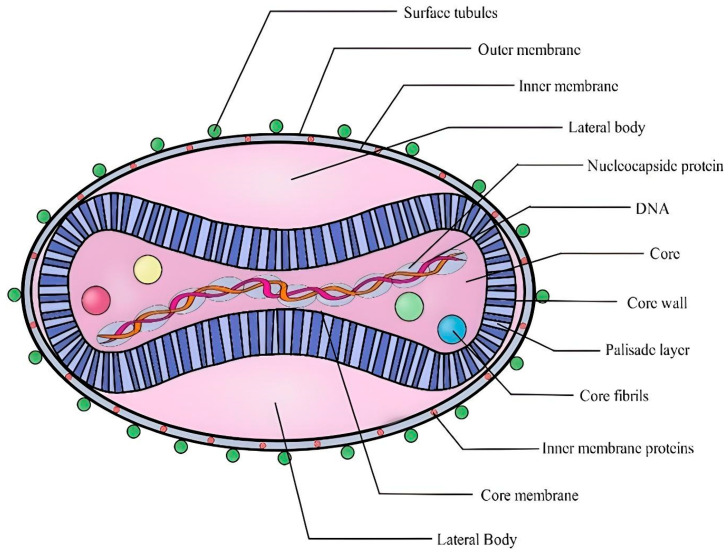
Structural features of a poxvirus virion. The virion is encased in an outer membrane and inner membrane, with surface tubules projecting from the outer surface. Inside, the core contains viral DNA, nucleocapsid proteins, and lateral bodies, surrounded by a core wall and fibrils. The palisade layer provides structural integrity, while inner membrane proteins facilitate viral assembly and infection. This unique design supports the virus’s ability to replicate and evade host defenses.

The mpox virus, along with other poxviruses, is one of the biggest and most complicated illnesses. The mpox virus is large enough to be visible under a fluorescence microscope and has an ultrastructure that can be viewed under an electron microscope. These are brick-like particles with widths ranging from 140 nm to 260 nm with an average particle size of 220 nm to 450 nm [58]. Hence, to differentiate the ultrastructure, the stronger amplification afforded by electron microscopy is required. The core, lateral bodies, outer membrane, and outer lipoprotein envelope are the four basic components of the orthopox virion [59]. The center core, encircled by a dense layer of rod-shaped structures known as the palisade layer, contains the viral double-stranded DNA (dsDNA) and core fibrils. The palisade layer, lateral bodies, and central core are enclosed by the outer membrane, which is composed of many surface tubules (Figure 2). In contrast to virions released by cellular rupture, spontaneously generated virions typically contain the outer lipoprotein membrane [60]. A mature virion seems to have around 80 viral proteins [61].

## 9. mpox Virus Genome

One of the largest viral genomes, the mpox genome is a gigantic 197 kb single linear dsDNA molecule [7]. A collection of brief tandem repeats [62], terminal hairpin loops [63], and identical but oppositely oriented terminal reads of around 6 kbp are present at every end of the genome. About 190 non-overlapping open-reading frames (>180 bp long), each containing 60 or perhaps more amino acid residues, make up the genome. The inverted terminal repeats exhibit 4 of these 190. mpox virus DNA contains only around 31.1% guanine and cytosine [29]. mpox virus has two separate genetic clades, the West African (WA) clade as well as the Central African (CA) clade [64].

**Figure 3:**
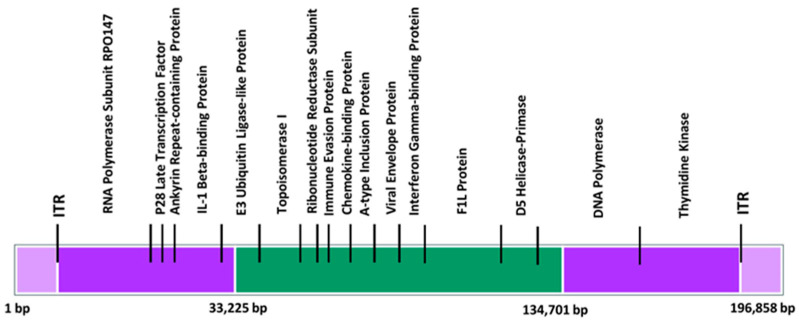
Genomic organization of the mpox virus, highlighting the inverted terminal repeats (ITRs) and conserved regions. The genome is depicted with purple regions at both ends representing variable regions flanked by ITRs. The central conserved region, shown in green, spans positions 33,225 bp to 134,701 bp. Various genes, including those labeled A to P, are marked along the genome, indicating their relative positions. This schematic highlights the structural organization and division between conserved and variable genomic regions in the mpox virus, providing insight into its genetic composition and potential functional domains.

It consists of inverted terminal repeats (ITR) of 10 kb at either end (Figure 3). Genes are densely packed; intergenic areas longer than 100 bp are uncommon. The center conserved area contains genes that perform” housekeeping” functions like transcription, replication, and virion assembly. The genes expressed in the terminal sections of poxviruses differ from one another and encode proteins implicated in the host range and pathogenicity [65]. Deep sequencing technology advancements have offered important insights into the standing nucleotide variation (SNV) inherent in poxvirus populations. These variants were previously unappreciated but are now recognized as major contributors to poxvirus evolution. Furthermore, there is increasing knowledge of the role that genomic architectural alterations play in determining poxvirus evolution. Key drivers of genomic architectural alterations in poxviruses have been identified as homologous and non-homologous recombination, gene duplications, gene loss, and horizontal gene transfer. A recent study [35] found a higher-than-expected incidence of SNV accumulation in recent samples of MPXV. Seven SNVs have been seen since the initial epidemic in March 2022, with a total of fifty SNVs discovered since 2018 [66]. The increased rate of SNV acquisition in recent MPXV isolates may be due to the host adaptability, effective population size, and selection coefficient. Selective sweeps or bottlenecks within individuals or during transmission may limit the accumulated diversity, leading to disparities between epidemiological isolates and phylogenetic data [67]. The intergenic region (ITR) of MPXV has been observed to expand or decrease, with varying clades having varying ITR lengths. Variants in the Z-encoding region of the virus, such as the E3L gene, have been found, potentially affecting PKR. Microsatellite variants, particularly the A26 ortholog, contribute to genetic variability and influence virus–host interactions. Overall, the data highlight the complexities of poxvirus evolution and the various pathways involved in producing genetic variation within poxvirus populations [68].

## 10. mpox Virus Replication Cycle

Poxvirus replication is distinct from that of other DNA viruses in that it occurs in the cytoplasm of the host cell. Poxviruses enter cells by an intricate mechanism that includes an attachment, hemi fusion, and core penetration within the plasma membrane, or via endocytosis [69]. The specific method that poxviruses employ to enter cells is determined by their infectious form, which may either be a mature virion (MV) through one outer membrane or an extracellular enveloped virion (EV) with a different protein composition. In the EV form, the membrane that is particularly EV-specific is eliminated, preserving the underlying membrane affixed to the cell. EV is more specialized for cell-to-cell transmission despite MV’s greater prevalence because of its long, mobile projections made by the polymerization of actin which adheres to the cell’s surface. When a mature virion enters a cell, it loses its protective coating. Once within the cell’s cytoplasm, the virus releases ready-made enzymes and certain other enzymatic components that weaken the cell’s defenses and start the synthesis of its original genes.

**Figure 4:**
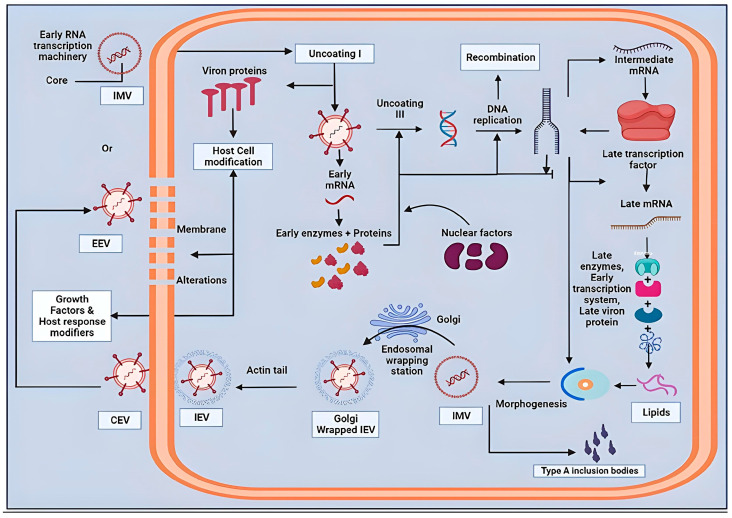
Replication cycle of poxvirus. The schematic representation illustrating the key stages in the viral lifecycle. The process begins with the entry of intracellular mature virions (IMVs) or extracellular enveloped virions (EEVs) into the host cell. Following uncoating, viral DNA is released into the cytoplasm for early mRNA transcription, leading to the synthesis of early proteins. DNA replication occurs in viral factories, followed by intermediate and late transcription for structural protein production. Morphogenesis leads to virion assembly, forming IMVs and EEVs. Host cell modifications, including actin tail formation and membrane alterations, facilitate viral egress. Type A inclusion bodies and Golgi involvement are highlighted.

Then, the early messenger RNA is made via the viral DNA-dependent polymerase (mRNA). The uncoating process that follows DNA replication and the creation of intermediary transcriptional regulators are both supported by early mRNA translation. The intermediate mRNA is then translated and expressed in late mRNAs, where it is converted into structural and non-structural proteins. Then, translated proteins and DNA concatemers generated during the initial stages of replication are assembled. They then transform, becoming immature virions, which mature into intracellular viruses (IMVs). Since IMVs are devoid of an outer membrane, they can only spread when cells are damaged. Intracellular enveloped virions (IEVs) are formed from IMV particles that do not wrap completely in the cytoplasmic protein matrix. These cells are aided in reaching the cell membrane by microtubules. They then combine with the cell membrane to generate cell-associated viruses (CEVs), that lead to actin polymerization and the production of filaments, which help to facilitate CEV evacuation from the cell. Both extracellular and intracellular viral pathogens have a significant impact on pathogenesis (Figure 4). The primary means of disease transmission from one cell to the next are intracellular viral virions (IMVs and IEVs), together with CEVs. EEVs are necessary for viral spread within an affected organism [70].

## 11. Signs and Symptoms of mpox Viral Infection

mpox is a self-limiting condition that typically disappears in two to four weeks. The three distinct stages of a less severe form of smallpox include incubation, prodrome, and rash, which are either indicators or symptoms of MPV infection. On average, the incubation phase lasts between seven and fourteen days [71]. Fever and lymphadenopathy are common during the prodrome phase, with the latter characterizing mpox as opposed to smallpox and chickenpox1. Compared to many other illnesses, such as the flu, mpox does not initially present with any noteworthy symptoms. In addition to other symptoms, patients may also have a fever, headache, muscular pains, and enlarged lymph nodes. A rash forms after a few days, first appearing on the face before moving to other parts of the body. The rash develops in a specific manner, beginning as a macular rash and continuing through popular, vesicular, and pustular phases before crusting over and flaking off. The rash often affects the face, trunk, and limbs, but it may also affect other parts of the body, such as the genitalia [71].

mpox symptoms are comparable to smallpox symptoms while being less severe, and most patients recover completely. With a mortality rate between 1 and 10%, mpox can be risky. How severe the infection is will depend on many factors, including the virus strain, the accessibility of medical care, the level of exposure, and the patient’s condition [72]. MPXV enters the body and replicates, spreading to lymph nodes, causing primary and secondary viremia. The virus primarily affects the skin, causing rash development, oral mucous membranes, genitalia, and conjunctivae. Anogenital lesions, pharyngitis, and lymphadenopathy are common symptoms. MPXV can also affect the respiratory system, leading to severe breathing difficulties, pneumonia, and lung inflammation. Pulmonary surfactants and lubricants can aid lung function. Oral cavity lesions, such as glossitis, stomatitis, and gingivitis, can spread. Lymphadenopathy is a distinguishing characteristic of MPX, manifesting as lymph node enlargement during primary and secondary viremia. MPXV causes relatively few liver lesions, but viral antigens can disseminate throughout the liver, leading to hepatocyte degeneration and liver lesions.

A recent journal paper [73] reports the clinical presentation of the first recorded case of MPXV, SARS-CoV-2, and HIV-1 co-infection. mpox and COVID-19 [51,52] are both airborne viruses that cause symptoms such as fever, lymphadenopathy, headache, sore throat, and exhaustion. This case study’s subject was a 36-year-old man who developed symptoms after visiting Spain. The patient tested positive for SARS-CoV-2 and had skin lesions similar to mpox. Co-infection with MPXV, SARS-CoV-2, and HIV- 1 was verified by diagnostic testing. The case illustrates the overlapping symptoms of mpox and COVID-19, emphasizing the significance of collecting a complete history and considering sexual behaviors for a correct diagnosis. Sexual contact appears to be a significant mechanism of transmission for mpox, necessitating extensive STI screening. Because the patient’s HIV-1 status indicates recent infection, care should be maintained even after clinical remission. Because there is presently no widely approved therapy or prevention for mpox, healthcare systems must be aware of the likelihood of co-infections and advocate adequate diagnostic testing in high-risk people.

## 12. Sampling and Diagnosis of mpox Virus

The PCR test is crucial for detecting the MPXV in suspected cases. Samples should be collected from exposed lesions, vesicles, or ulcers, with pharyngeal swabs taken if throat lesions are present. Proper labeling is essential in order to avoid processing errors. Healthcare workers must wear personal protective equipment (PPE), including droplet, contact, and eye protection, during diagnostic procedures to ensure safety. For high-risk cases without visible lesions, throat swabs may be used, with continued symptom monitoring if results are negative. Samples should be transported to local or advanced laboratories for MPXV testing, alongside tests for other infections like HSV or syphilis. Follow-up testing for confirmed cases includes lesion and throat swabs, blood samples in EDTA tubes, and urine samples, all stored in appropriate mediums. These measures provide valuable insights into disease progression and inform decisions on transitioning patients from isolation to inpatient care [74].

Accurate diagnostic testing for mpox requires proper sampling and storage techniques. Specimens should be placed in sterile, leak-proof containers suitable for shipping under refrigerated (2–8 °C) or frozen (−20 °C or lower) conditions. Dry swabs, lesion crusts, and swabs in a viral transport medium (VTM) can be tested within 7 days if refrigerated, or within 30–60 days if frozen, depending on the specimen type. For shipping, specimens must be categorized as Biological Substances, bagged individually to prevent rejection due to leaks, and transported on dry ice to maintain the required temperature. Packaging must ensure structural integrity and allow for gas release from dry ice. Only trained personnel should handle hazardous shipments, which must be labeled with all necessary identifiers and contact details [75]. These protocols are critical for safely transporting mpox samples linked to Clade II (the West African clade) for laboratory testing. Diagnostic results must be correlated with clinical and epidemiological data to confirm mpox infection. Following these guidelines ensures specimen integrity and accurate testing. To further confirm mpox virus infection, diagnostic tests, and clinical and epidemiological data must be correlated. The diagnosis of mpox (Table 2) virus infection is based on clinical signs, laboratory tests, and medical history [53]. By checking for lymphadenopathy at the prodromal stage of the illness, it is possible to clinically distinguish mpox from chickenpox or smallpox [76].

The specimen samples are collected and forwarded to the lab to verify the diagnosis in compliance with the WHO criteria. PCR is the most widely used laboratory diagnostic method to identify mpox due to its accuracy and sensitivity [78]. The fluid that is produced by vesicles and pustules, as well as dry crusts are the best samples to use in the diagnosis of mpox. When a lesion sample needs to be kept cool and kept in a dry, sterile tube without a viral transport medium, a biopsy appears to be an alternative in some circumstances. PCR blood tests are often inconclusive and should not be routinely obtained from patients because viremia typically lasts for a short period compared to the time the material is taken after symptoms start [79]. The recommended specimen type for diagnosing mpox is skin lesion material, including swabs of lesion exudate, roofs from multiple lesions, or lesion crusts. Testing should be offered to individuals meeting the suspected case definition, considering both clinical and epidemiological factors. It can be challenging to differentiate mpox based solely on clinical presentation, and other potential causes of skin lesions should be considered. Safety procedures, including proper personal protective equipment and handling of specimens, must be followed. The collection and storage of specimens, including skin lesion material and additional types for research purposes, are needed.

Nucleic acid amplification testing (NAAT), particularly polymerase chain reaction, is recommended for confirming mpox infection. Reagents, controls, waste disposal, and biosafety measures should be appropriately managed during testing. Serological testing and genetic sequencing are additional tools for diagnosis and surveillance. Overall, the adherence to biosafety measures and risk assessments are crucial in handling specimens and conducting laboratory testing for mpox. For the confirmation of MPXV infection, clinical and epidemiological data should be considered [80]. Positive detection using an orthopoxvirus (OPXV) polymerase chain reaction (PCR) assay, followed by MPXV confirmation via PCR and/or sequencing, or, in suspected cases, positive detection using an MPXV-specific PCR, confirms MPXV infection. Although MPXV-specific confirmatory testing is preferred, a positive OPXV PCR assay result is sufficient for laboratory confirmation. In instances where the clinical presentation and epidemiology indicate MPXV infection despite negative PCR results, serological testing can be utilized to investigate a prior infection. False-negative results can be influenced by factors such as specimen quality, handling, transportation, and technical issues [81]. Genetic sequence data (GSD), obtained through sequencing positive MPXV specimens, can provide valuable information on the virus’s origins, epidemiology, and characteristics. The WHO encourages countries and laboratories to share GSD, including raw data, through public access databases to enhance understanding and collaboration. Sequencing can be performed using Sanger or next-generation sequencing methods [82]. Laboratories must adhere to national reporting regulations. Positive or negative test findings should be immediately reported to national authorities. States Parties to the IHR are reminded of their responsibility to communicate relevant public health information with the WHO for occurrences that they have notified the WHO. Access to the quick and reliable laboratory testing of samples from cases under investigation is critical for diagnosing and monitoring this developing virus. All nations should have access to reliable testing, either locally or by referral to laboratories in other countries willing and capable of diagnosing OPXV or MPXV. The WHO’s Regional Offices can help Member States gain access to testing through referral. The inactivation of samples at a local laboratory, where suitable and securely conducted, may improve referral and alleviate logistical issues. Countries are invited to exchange their sequencing data so that the present outbreak can be better understood.

## 13. MPXV and Mechanisms of Immune Evasion

MPXV has sequence similarities with the vaccinia virus (VACV) and uses a variety of strategies to avoid detection by the immune system. The MPXV F3 protein and the VACV E3 protein are homologous, indicating a possible functional relationship. MPXV suppresses the antiviral immune response more effectively than a VACV mutant missing the *N*-terminal region of its E3 homolog. This suggests that MPXV may prevent symptoms caused by its E3 homolog’s lack of the N-terminal region. MPXV cannot downregulate MHC Class I; instead, it employs a method to prevent CD4+ and CD8+ T-cell activation upon interaction with MPXV-infected cells [83]. By suppressing local T-cell responses, MPXV can evade the immune system while maintaining a viral reservoir. MPXV-encoded immunomodulators play a critical role in blocking antiviral T-cell responses, which are activated by the host. Neutralizing antibodies are crucial for the protection against severe MPXV infections, while memory T-cells alone do not provide sufficient protection [83].

The spread of MPXV relies on interactions with circulating monocytes, which may protect the virus from humoral immune responses. Macrophages and other immune cells may also aid in the spread of MPXV. Understanding the immune evasion mechanisms of MPXV and the interactions between various orthopoxviruses, such as VACV, is important for studying immunity to mpox and developing effective preventive measures like vaccinations [84].

## 14. Treatment for mpox

Fortunately, the clinical course of mpox infections is usually mild and self-limiting. As a result, it infrequently justifies specialized therapy, and, instead, treatment is typically supportive. Supportive therapy includes things like analgesics for pain, antipyretics for fever, and antibiotics for subsequent bacterial infections. Yet, some people could require a certain kind of treatment. For those with serious illnesses, immunocompromised people, pregnant women, and youngsters, specific therapies can be required [85,86]. The treatment and immunizations created to combat smallpox have shown some success against the mpox virus because of the similarities between mpox and smallpox. There is, however, little evidence to support this claim [87].

mpox disease is not curable; however, research on treating smallpox indicates that vaccinia vaccinations, tecovirimat, vaccinia immune globulin (IVG), and cidofovir might be beneficial [88]. Cidofovir is an antiviral drug that inhibits the viral DNA polymerase and exerts antiviral activity against a variety of viruses. It is usually administered intravenously with fluid administration and probenecid. Although cidofovir has the potential to cause nephrotoxicity (kidney toxicity), a modified version called CMX-001 has been developed that is not nephrotoxic. In addition, CMX-001 exhibits antiviral activity against numerous orthopoxvirus species. ST-246 is an agent that is administered orally and targets the intracellular release of the virus. It has shown promising results against several orthopoxviruses, including the variola virus that causes smallpox. By inhibiting the release of viral particles from infected cells, ST-246 helps to contain the spread of infection. The European Medicines Agency (EMA) authorized the usage of tecovirimat for treating measles in 2022, reported by the World Health Organization [89]. It was first created as a smallpox remedy. Because tecovirimat is currently not widely available, any usage of it needs to be carefully supervised. By blocking the viral DNA polymerase, cidofovir has antiviral efficacy against some viruses [90]. It has been observed that tecovirimat is particularly sensitive to several orthopoxviruses, including variola, cowpox, vaccinia, rabbitpox, ectromelia, and mpox. For the treatment of mpox, tecovirimat, an oral inhibitor of intracellular viral release, may be beneficial [91,92]. These antiviral agents have been studied in various combinations, such as with vaccinia immunoglobulin, which is used to treat serious adverse events associated with smallpox vaccination clinical trials. The combination of these drugs has the potential to effectively treat orthopoxvirus infections. Notwithstanding the recommended course of treatment, supportive and symptomatic therapy is the backbone of treating a mpox virus infection.

It is crucial to realize that the only established treatment for mpox is treating symptoms and preventing complications. Given the outbreak of mpox in 2022 and the current mpox cases worldwide, more research must be carried out before any treatment or vaccine can be created [93]. To prevent infection with the mpox virus, certain precautions can be taken. This includes, but is not limited to, the following: (1) consciously avoiding encounters with animals suspected to be mpox virus carriers, especially in regions where the mpox virus is prevalent; (2) placing ill people in a room with negative pressure to prevent the virus from spreading from one person to another; (3) isolating and euthanizing the animals suspected to be the virus reservoirs; (4) avoiding contact with any objects that have come into contact with ill animals or people; and (5) avoiding contact with ill people. Frontline personnel caring for mpox virus-infected patients and other high-risk individuals who are expected to interact with infected individuals should wear the appropriate personal protective equipment (PPE) designed to protect against airborne infectious agents, such as N-95 masks, fully covered water-resistant gowns, and double-layered gloves [94,95].

## 15. Vaccination/Prevention of mpox

Research has shown that smallpox vaccination offers a reliable defense against illnesses caused by mpox and other orthopoxviruses [96]. When administered early in the incubation phase, it can postpone the start of the disease or minimize the severity of the illness. However, patients who have impaired immune systems face the risk of suffering serious side effects [97,98]. mpox has become more prevalent since smallpox was eradicated in 1980 as a result of the end of immunization initiatives to prevent viral illnesses.

Several vaccines are available for preventing mpox, each with distinct mechanisms and efficacy levels, playing a critical role in responding to recent outbreaks, particularly in non-endemic regions in 2022. JYNNEOS (Imvamune/Imvanex) is a non-replicating modified vaccinia Ankara (MVA) vaccine developed by Bavarian Nordic. Widely used for preventing smallpox and mpox, especially in high-risk populations, it has demonstrated safety and efficacy in clinical trials. Strong immune responses have been observed in healthy adults and individuals with HIV. Approved by the FDA for adults, JYNNEOS has shown excellent outcomes in preventing severe disease among healthcare workers and close contacts of infected individuals. Its safety profile, particularly for immunocompromised individuals, and its availability in countries like the U.S., Canada, and the EU make it a cornerstone in mpox prevention [99,100]. ACAM2000, produced by Emergent BioSolutions, is a live, replication-competent smallpox vaccine authorized under the FDA’s Emergency Use Authorization (EUA) for mpox. While highly effective in preventing smallpox, its live-virus composition increases the risks of side effects, including myocarditis and complications in immunocompromised individuals. Despite these risks, ACAM2000 remains a valuable option in outbreak scenarios [101]. LC16m8, developed by KM Biologics, is a live attenuated smallpox vaccine with WHO emergency use listing. Administered via a bifurcated needle, it is effective as a single-dose vaccine for individuals aged one year and older. Widely used in low-resource settings, it provides strong protection against mpox and smallpox with fewer side effects than ACAM2000. Its affordability and ease of administration make it particularly valuable in endemic regions like Africa [100]. mRNA Vaccines, exemplified by Moderna’s mRNA-1769, represent a promising new platform for mpox prevention. Currently in Phase I/II trials, this vaccine is designed to protect against both smallpox and mpox. Early results indicate safety and robust immune responses. If successful, mRNA-1769 could provide a flexible, efficient approach to combating mpox, leveraging the same mRNA technology used effectively in COVID-19 vaccines [102]. These vaccines, with unique characteristics and clinical results, are integral to global mpox control efforts. Their availability, coupled with public health interventions such as vaccination campaigns, contact tracing, and health education, is essential to mitigating outbreaks. The advancement of vaccine technologies, particularly mRNA platforms, holds significant promise for enhancing our preparedness against mpox and other emerging infectious diseases. By leveraging these tools and strategies, the global health community can better respond to current and future outbreaks, ultimately reducing mpox’s burden worldwide.

The attenuated strains used in the next-generation smallpox vaccines, including ACAM2000 (this is a live vaccinia virus), LC16m8 (this one is an attenuated vaccinia virus), and modified vaccinia Ankara (this is also an attenuated vaccinia virus), not only have a better safety profile than the first- and second-generation smallpox vaccines, but also successfully stimulate the production of antibodies in atopic and immunocompromised patients [16,103,104,105]. The live smallpox vaccination ACAM2000 has the benefit of being delivered in a single dosage. This medication is not recommended since it can proliferate in the cells of immunocompromised people. Furthermore, pregnant women and anyone with eczema or atopic dermatitis should not be vaccinated. A small number of vaccinated people may experience cardiac problems. Increased lesioning at the location of immunization is normal and should be expected. The LC16m8 vaccine is an attenuated smallpox virus vaccine.

IMVAMUNE, a modified live attenuated vaccine, has fewer adverse effects and a safer profile than ACAM2000. Known as IMVAMUNE in the US and IMVANEX in Europe, it has a limited replication ability, making it suitable for all adults, including those with weakened immune systems. Even after two doses of the live ACAM2000 vaccine, no skin lesions appear at the vaccination site [106]. Given the global impact of the mpox virus (MPV), particularly in the context of the COVID-19 era, it is imperative that national and international research efforts be intensified. These efforts should focus on identifying virulence markers of the disease, understanding the factors that influence MPV evolution, which include both host and viral factors, investigating human behaviors that contribute to zoonotic spillover events, finding surrogates for asymptomatic infections, and examining the determinants of immunity for both the virus and the host. The goal of this research is to prevent MPV from filling the ecological gap left by the eradication of the variola major virus (VARV) and potentially evolving into a more dangerous pathogen. To achieve this, the preventive epidemiological surveillance of MPV in endemic and high-risk areas, particularly in Nigeria, is essential. This surveillance should be conducted routinely and not only in response to outbreaks. Support the inclusion of routine and regular epidemiological surveillance of MPV in humans and animals in existing surveillance systems, such as the Surveillance Outbreak Response Management and Analysis System (SORMAS). This would allow the early detection and effective management of mpox epidemics [107]. The Sewer Coronavirus Alert Network (SCAN) was established in 2020 to test wastewater samples for the presence of the COVID-19 virus. Since June 2022, it has expanded to include MPXV detection in water plants. Combining this network with a phylogenomic analysis could provide insights into MPXV’s spread and evolution. Although criticized for being reactive, SCAN has proven effective in anticipating the virus spread, enabling frontline workers to control outbreaks and prevent a potential new mpox pandemic.

## 16. Recent Advances in mpox Clinical Trials

The JYNNEOS vaccine, approved for smallpox and mpox prevention, has been central to clinical efforts. Recent trials have explored alternative dosing strategies, such as intradermal administration, to expand the vaccine supply. This method uses one-fifth of the standard subcutaneous dose, enabling healthcare providers to vaccinate more people without compromising immunogenicity. Initial findings indicate comparable immune responses and safety profiles, making this approach vital in public health emergencies [108]. Other vaccine candidates, like ACAM2000 and LC16m8, have also undergone evaluations. ACAM2000, a replication-competent smallpox vaccine, has been authorized under emergency use for mpox. Despite its efficacy, its side effects, such as myocarditis risks, limit widespread use. LC16m8, a live attenuated vaccine, has emerged as a safer alternative, especially for immunocompromised individuals. Studies confirm its effectiveness as a single-dose vaccine, particularly in endemic regions [109]. Additionally, efforts to innovate mpox vaccines using mRNA technology are underway. Moderna is conducting early-phase trials for its mRNA-1769 vaccine, aiming to offer a flexible platform similar to COVID-19 vaccines. These trials seek to establish safety and robust immune responses, with large-scale studies planned in the future [110]. Complementing these efforts, the National Institutes of Health (NIH) and the Centers for Disease Control and Prevention (CDC) are conducting serological studies to track mpox transmission and evaluate vaccine effectiveness. These initiatives help refine outbreak responses and improve future preparedness.

In terms of therapeutics, the antiviral drug tecovirimat (TPOXX) has been a focus of several studies. This drug, initially approved for smallpox, has shown promise in treating mpox. Randomized controlled trials in the U.S. and the Democratic Republic of Congo are assessing its safety and efficacy for mpox treatment. The trials include diverse populations, including immunocompromised individuals and pregnant women, to gather comprehensive safety data [111]. However, recent studies revealed that studies on tecovirimat in the Democratic Republic of the Congo and by the NIH found the antiviral safe but ineffective in improving mpox symptom resolution or pain reduction, including for Clade I cases. These findings highlight the need for further research to optimize mpox treatments [112,113].

## 17. Conclusions and Future Directions for mpox Research and Control Measures

Indeed, the mpox virus has long been a neglected zoonotic disease with the potential to spread and/or be used as a bioweapon. There are now no traditional guidelines for clinical care, medications, or vaccinations, although the condition was originally diagnosed in 1958 and first recorded in a human in 1970 [114]. The limited research on the human immune response to MPXV infection remains unanswered. Understanding immune defenses and the potential for mucosal protection resulting from smallpox immunization or MPXV infection is essential. Investigating tissue-resident memory T cells and IgA in MPXV-related respiratory complications is essential. Factors like behavior, geography, diet, medical history, immunological factors, and genetics may also impact MPXV susceptibility and immunity. The virus can spread globally and has the potential to expand beyond Africa. Effective management strategies, such as ring-vaccination and pre-exposure vaccination, are needed to combat the outbreak. Increased surveillance efforts, vaccination availability, and antiviral medications can help mitigate the threat of MPXV. Moreover, it is a clear call to action for public health authorities to assume responsibility for informing specialists in the field of their results and being open and truthful with the general public. Recent outbreaks around the globe have highlighted the significance of rigorous and ongoing surveillance, as well as the creation of novel preventative and therapeutic strategies. To stop rising transmission rates or virulence, appropriate and effective medicines as well as active monitoring activities are urgently needed [78]. At least in endemic regions and possibly globally, the mpox virus is the predominant orthopoxvirus in humans. It takes a lot of effort to prevent mpox, which is no longer a rare condition. The human mpox virus should be monitored and controlled using the insights learnt from the COVID-19 pandemic.

## Figures and Tables

**Table 1:** Orthopoxvirus classification.

Family	Poxviridae
Subfamily	Chordopoxvirinae
Genus	*Orthopoxvirus*
Species	*Orthopoxvirus monkeypox*
	(including camelpox virus, cowpox virus, Ectromelia virus, raccoonpox virus, skunkpox virus, taterapox virus, vaccinia virus, variola virus, and vVolepox virus)

**Table 2:** Updated diagnostic tests for mpox. Table modified from Chemma et. al. [77].

Tests	Description	Sample Used
PCR	It is based on NAAT; the gold standard for detecting mpox DNA is presently real-time PCR.	Lesion fluid
Viral culture	From a patient sample, the virus is cultured and isolated.	Lesion fluid
Electron microscopy	Using an electron microscope, pox viruses are morphologically identified.	Biopsy specimen, scab material, vesicular fluid
Immunohistochemistry	Antigens specific to the orthopoxvirus are evaluated for their presence.	Biopsy specimen
Anti-orthopoxvirus IgG and IgM tests	These tests may be used to determine if you have been exposed to orthopoxvirus recently or in the past.	Blood specimen

PCR, polymerase chain reaction; NAAT, nucleic acid amplification test; DNA, deoxyribonucleic acid; IgG, immunoglobulin G; IgM, immunoglobulin M.

## Data Availability

The data are available upon request.

## References

[1] WHO (2024). WHO Director-General Declares Mpox Outbreak a Public Health Emergency of International Concern.

[2] McCollum A.M., Damon I.K. (2014). Human monkeypox. Clin. Infect. Dis..

[3] Bunge E.M., Hoet B., Chen L., Lienert F., Weidenthaler H., Baer L.R., Steffen R. (2022). The changing epidemiology of human monkeypox-A potential threat? A systematic review. PLoS Negl. Trop. Dis..

[4] Islam M.M., Dutta P., Rashid R., Jaffery S.S., Islam A., Farag E., Zughaier S.M., Bansal D., Hassan M.M. (2023). Pathogenicity and virulence of monkeypox at the human-animal-ecology interface. Virulence.

[5] Kumar S., Subramaniam G., Karuppanan K. (2023). Human monkeypox outbreak in 2022. J. Med. Virol..

[6] Parker S., Buller R.M. (2013). A review of experimental and natural infections of animals with monkeypox virus between 1958 and 2012. Future Virol..

[7] Kmiec D., Kirchhoff F. (2022). Monkeypox: A New Threat?. Int. J. Mol. Sci..

[8] Ligon B.L. (2004). Monkeypox: A review of the history and emergence in the Western hemisphere. Semin. Pediatr. Infect. Dis..

[9] Cabanillas B., Valdelvira R., Akdis C.A. (2022). Monkeypox outbreak in Europe, UK, North America, and Australia: A changing trend of a zoonotic disease. Allergy.

[10] Gigante C.M., Korber B., Seabolt M.H., Wilkins K., Davidson W., Rao A.K., Zhao H., Smith T.G., Hughes C.M., Minhaj F. (2022). Multiple lineages of monkeypox virus detected in the United States, 2021–2022. Science.

[11] Gould S., Atkinson B., Onianwa O., Spencer A., Furneaux J., Grieves J., Taylor C., Milligan I., Bennett A., Fletcher T. (2022). Air and surface sampling for monkeypox virus in a UK hospital: An observational study. Lancet Microbe.

[12] Nguyen P.Y., Ajisegiri W.S., Costantino V., Chughtai A.A., MacIntyre C.R. (2021). Reemergence of Human Monkeypox and Declining Population Immunity in the Context of Urbanization, Nigeria, 2017–2020. Emerg. Infect. Dis..

[13] Grant R., Nguyen L.L., Breban R. (2020). Modelling human-to-human transmission of monkeypox. Bull. World Health Organ..

[14] Petersen E., Kantele A., Koopmans M., Asogun D., Yinka-Ogunleye A., Ihekweazu C., Zumla A. (2019). Human Monkeypox: Epidemiologic and Clinical Characteristics, Diagnosis, and Prevention. Infect. Dis. Clin. N. Am..

[15] Kumar S., Karuppanan K., Subramaniam G. (2022). Omicron (BA.1) and sub-variants (BA.1.1, BA.2, and BA.3) of SARS-CoV-2 spike infectivity and pathogenicity: A comparative sequence and structural-based computational assessment. J. Med. Virol..

[16] Pittman P.R., Hahn M., Lee H.S., Koca C., Samy N., Schmidt D., Hornung J., Weidenthaler H., Heery C.R., Meyer T.P.H. (2019). Phase 3 Efficacy Trial of Modified Vaccinia Ankara as a Vaccine against Smallpox. N. Engl. J. Med..

[17] Christodoulidou M.M., Mabbott N.A. (2023). Efficacy of smallpox vaccines against Mpox infections in humans. Immunother. Adv..

[18] Xiridou M., Miura F., Adam P., Op de Coul E., de Wit J., Wallinga J. (2024). The Fading of the Mpox Outbreak Among Men Who Have Sex With Men: A Mathematical Modelling Study. J. Infect. Dis..

[19] Allan-Blitz L.T., Gandhi M., Adamson P., Park I., Bolan G., Klausner J.D. (2023). A Position Statement on Mpox as a Sexually Transmitted Disease. Clin. Infect. Dis..

[20] Adalja A., Inglesby T. (2022). A Novel International Monkeypox Outbreak. Ann. Intern. Med..

[21] Dye C., Kraemer M.U.G. (2022). Investigating the monkeypox outbreak. BMJ.

[22] Martín-Delgado M.C., Martín Sánchez F.J., Martínez-Sellés M., Molero García J.M., Moreno Guillén S., Rodríguez-Artalejo F.J., Ruiz-Galiana J., Cantón R., De Lucas Ramos P., García-Botella A. (2022). Monkeypox in humans: A new outbreak. Rev. Esp. Quimioter..

[23] Minhaj F.S., Ogale Y.P., Whitehill F., Schultz J., Foote M., Davidson W., Hughes C.M., Wilkins K., Chatelain R., Donnelly M.A.P. (2022). Monkeypox Outbreak—Nine States, May 2022. MMWR Morb. Mortal. Wkly. Rep..

[24] Soheili M., Nasseri S., Afraie M., Khateri S., Moradi Y., Mahdavi Mortazavi S.M., Gilzad-Kohan H. (2022). Monkeypox: Virology, Pathophysiology, Clinical Characteristics, Epidemiology, Vaccines, Diagnosis, and Treatments. J. Pharm. Pharm. Sci..

[25] Zahmatyar M., Fazlollahi A., Motamedi A., Zolfi M., Seyedi F., Nejadghaderi S.A., Sullman M.J.M., Mohammadinasab R., Kolahi A.A., Arshi S. (2023). Human monkeypox: History, presentations, transmission, epidemiology, diagnosis, treatment, and prevention. Front. Med..

[26] Ramírez-Olivencia G., Velasco Arribas M., Vera García M.M., Casabona J., Martínez M.J., Membrillo De Novales F.J., Group C.S. (2024). Clinical and Epidemiological Characteristics of the 2022 Mpox Outbreak in Spain (CEME-22 Study). Open Forum Infect. Dis..

[27] Cuetos-Suárez D., Gan R.K., Cuetos-Suárez D., Arcos González P., Castro-Delgado R. (2024). A Review of Mpox Outbreak and Public Health Response in Spain. Risk Manag. Healthc. Policy.

[28] Gessain A., Nakoune E., Yazdanpanah Y. (2022). Monkeypox. N. Engl. J. Med..

[29] Shchelkunov S.N., Totmenin A.V., Safronov P.F., Mikheev M.V., Gutorov V.V., Ryazankina O.I., Petrov N.A., Babkin I.V., Uvarova E.A., Sandakhchiev L.S. (2002). Analysis of the monkeypox virus genome. Virology.

[30] Senkevich T.G., Yutin N., Wolf Y.I., Koonin E.V., Moss B. (2021). Ancient Gene Capture and Recent Gene Loss Shape the Evolution of Orthopoxvirus-Host Interaction Genes. mBio.

[31] Babkin I.V., Babkina I.N., Tikunova N.V. (2022). An Update of Orthopoxvirus Molecular Evolution. Viruses.

[32] Thézé J., Takatsuka J., Li Z., Gallais J., Doucet D., Arif B., Nakai M., Herniou E.A. (2013). New insights into the evolution of Entomopoxvirinae from the complete genome sequences of four entomopoxviruses infecting Adoxophyes honmai, Choristoneura biennis, Choristoneura rosaceana, and Mythimna separata. J. Virol..

[33] Desingu P.A., Rubeni T.P., Sundaresan N.R. (2022). Evolution of monkeypox virus from 2017 to 2022: In the light of point mutations. Front. Microbiol..

[34] Wang L., Shang J., Weng S., Aliyari S.R., Ji C., Cheng G., Wu A. (2023). Genomic annotation and molecular evolution of monkeypox virus outbreak in 2022. J. Med. Virol..

[35] Isidro J., Borges V., Pinto M., Sobral D., Santos J.D., Nunes A., Mixão V., Ferreira R., Santos D., Duarte S. (2022). Phylogenomic characterization and signs of microevolution in the 2022 multi-country outbreak of monkeypox virus. Nat. Med..

[36] Baptista-Hon D.T., Fesalbon G.J.W., Monteiro O. (2022). Changing clinical features of the 2022 monkeypox global health emergency. MedComm–Future Med..

[37] Strathdee S.A., Smith D.M., Halbrook M., Mbala-Kingebeni P., Abeles S., Torriani F., Rimoin A. (2022). The rapidly evolving monkeypox epidemic: A call to action to leave no one behind. PLoS Med..

[38] Zhu J., Yu J., Qin H., Chen X., Wu C., Hong X., Zhang Y., Zhang Z. (2023). Exploring the key genomic variation in monkeypox virus during the 2022 outbreak. BMC Genom. Data.

[39] Chen N., Li G., Liszewski M.K., Atkinson J.P., Jahrling P.B., Feng Z., Schriewer J., Buck C., Wang C., Lefkowitz E.J. (2005). Virulence differences between monkeypox virus isolates from West Africa and the Congo basin. Virology.

[40] Dinarello C.A. (2018). Overview of the IL-1 family in innate inflammation and acquired immunity. Immunol. Rev..

[41] Weaver J.R., Isaacs S.N. (2008). Monkeypox virus and insights into its immunomodulatory proteins. Immunol. Rev..

[42] Ndodo N., Ashcroft J., Lewandowski K., Yinka-Ogunleye A., Chukwu C., Ahmad A., King D., Akinpelu A., Maluquer de Motes C., Ribeca P. (2023). Distinct monkeypox virus lineages co-circulating in humans before 2022. Nat. Med..

[43] Azzi A. (2023). Unusual Monkeypox virus outbreak in 2022: Phenotypic and molecular characteristics. Asp. Mol. Med..

[44] Nalca A., Rimoin A.W., Bavari S., Whitehouse C.A. (2005). Reemergence of monkeypox: Prevalence, diagnostics, and countermeasures. Clin. Infect. Dis..

[45] Katsafanas G.C., Moss B. (2007). Colocalization of transcription and translation within cytoplasmic poxvirus factories coordinates viral expression and subjugates host functions. Cell Host Microbe.

[46] WHO (2023). Multi-Country Monkeypox Outbreak: Situation Update.

[47] WHO (2024). Multi-Country Outbreak of Mpox.

[48] National Institute for Communicable Diseases (2024). Mpox Outbreak Alert: Africa’s Crisis and South Africa’s Response.

[49] WHO (2024). Mpox—Democratic Republic of the Congo.

[50] The United Nations Population Fund (2024). Central African Republic Situation Report #8–August 2024.

[51] Dou Y.M., Yuan H., Tian H.W. (2023). Monkeypox virus: Past and present. World J. Pediatr..

[52] Iñigo Martínez J., Gil Montalbán E., Jiménez Bueno S., Martín Martínez F., Nieto Juliá A., Sánchez Díaz J., García Marín N., Córdoba Deorador E., Nunziata Forte A., Alonso García M. (2022). Monkeypox outbreak predominantly affecting men who have sex with men, Madrid, Spain, 26 April to 16 June 2022. Eurosurveillance.

[53] Kumar N., Acharya A., Gendelman H.E., Byrareddy S.N. (2022). The 2022 outbreak and the pathobiology of the monkeypox virus. J. Autoimmun..

[54] Jairoun A.A., Al-Hemyari S.S., Abdulla N.M., El-Dahiyat F., Shahwan M., Hassan N., Jairoun O., Alyousef N.G., Sharif S., Jaber A.A.S. (2022). Awareness and preparedness of human monkeypox outbreak among university student: Time to worry or one to ignore?. J. Infect. Public Health.

[55] Kozlov M. (2022). Monkeypox outbreaks: 4 key questions researchers have. Nature.

[56] Vaughan A., Aarons E., Astbury J., Brooks T., Chand M., Flegg P., Hardman A., Harper N., Jarvis R., Mawdsley S. (2020). Human-to-Human Transmission of Monkeypox Virus, United Kingdom, October 2018. Emerg. Infect. Dis..

[57] Lansiaux E., Jain N., Laivacuma S., Reinis A. (2022). The virology of human monkeypox virus (hMPXV): A brief overview. Virus Res..

[58] Hyun J. (2022). Poxvirus under the eyes of electron microscope. Appl. Microsc..

[59] Buller R.M., Palumbo G.J. (1991). Poxvirus pathogenesis. Microbiol. Rev..

[60] Appleyard G., Hapel A.J., Boulter E.A. (1971). An antigenic difference between intracellular and extracellular rabbitpox virus. J. Gen. Virol..

[61] Resch W., Hixson K.K., Moore R.J., Lipton M.S., Moss B. (2007). Protein composition of the vaccinia virus mature virion. Virology.

[62] Wittek R., Moss B. (1980). Tandem repeats within the inverted terminal repetition of vaccinia virus DNA. Cell.

[63] Baroudy B.M., Venkatesan S., Moss B. (1982). Incompletely base-paired flip-flop terminal loops link the two DNA strands of the vaccinia virus genome into one uninterrupted polynucleotide chain. Cell.

[64] Likos A.M., Sammons S.A., Olson V.A., Frace A.M., Li Y., Olsen-Rasmussen M., Davidson W., Galloway R., Khristova M.L., Reynolds M.G. (2005). A tale of two clades: Monkeypox viruses. J. Gen. Virol..

[65] Yeh T.-Y., Hsieh Z.-Y., Feehley M.C., Feehley P.J., Contreras G.P., Su Y.-C., Hsieh S.-L., Lewis D.A. (2022). Recombination shapes 2022 monkeypox outbreak. Med.

[66] Brennan G., Stoian A.M.M., Yu H., Rahman M.J., Banerjee S., Stroup J.N., Park C., Tazi L., Rothenburg S. (2023). Molecular Mechanisms of Poxvirus Evolution. mBio.

[67] McGrail J.P., Mondolfi A.P., Ramírez J.D., Vidal S., García-Sastre A., Palacios G., Sanchez-Seco M.P., Guerra S. (2024). Comparative Analysis of 2022 Outbreak MPXV and Previous Clade II MPXV. J. Med. Virol..

[68] Brinkmann A., Kohl C., Pape K., Bourquain D., Thürmer A., Michel J., Schaade L., Nitsche A. (2023). Extensive ITR expansion of the 2022 Mpox virus genome through gene duplication and gene loss. Virus Genes.

[69] Moss B. (2012). Poxvirus cell entry: How many proteins does it take?. Viruses.

[70] Smith G.L., Vanderplasschen A., Law M. (2002). The formation and function of extracellular enveloped vaccinia virus. J. Gen. Virol..

[71] Xiang Y., White A. (2022). Monkeypox virus emerges from the shadow of its more infamous cousin: Family biology matters. Emerg. Microbes Infect..

[72] Thornhill J.P., Antinori A., Orkin C.M. (2022). Monkeypox Virus Infection across 16 Countries—April-June 2022. Reply N. Engl. J. Med..

[73] Nolasco S., Vitale F., Geremia A., Tramuto F., Maida C.M., Sciuto A., Coco C., Manuele R., Frasca E., Frasca M. (2023). First case of monkeypox virus, SARS-CoV-2 and HIV co-infection. J. Infect..

[74] UK Health Security Agency (2022). Mpox: Diagnostic testing.

[75] CDC (2024). Biosafety Laboratory Guidance for Handling and Processing Mpox Specimens.

[76] Moyo E., Musuka G., Murewanhema G., Moyo P., Dzinamarira T. (2022). Monkeypox outbreak: A perspective on Africa’s diagnostic and containment capacity. Int. J. Infect. Dis..

[77] Cheema A.Y., Ogedegbe O.J., Munir M., Alugba G., Ojo T.K. (2022). Monkeypox: A Review of Clinical Features, Diagnosis, and Treatment. Cureus.

[78] Paniz-Mondolfi A., Guerra S., Muñoz M., Luna N., Hernandez M.M., Patino L.H., Reidy J., Banu R., Shrestha P., Liggayu B. (2023). Evaluation and validation of an RT-PCR assay for specific detection of monkeypox virus (MPXV). J. Med. Virol..

[79] Stoykova Z., Kostadinova T., Todorova T., Niyazi D., Bozhkova M., Bizheva S., Stoeva T. (2022). Dealing with inconclusive SARS-CoV-2 PCR samples-Our experience. PLoS ONE.

[80] Zhou Y., Chen Z. (2023). Mpox: A review of laboratory detection techniques. Arch. Virol..

[81] WHO (2024). Diagnostic Testing for the Monkeypox Virus (MPXV).

[82] Altindis M., Puca E., Shapo L. (2022). Diagnosis of monkeypox virus—An overview. Travel. Med. Infect. Dis..

[83] Zandi M., Shafaati M., Hosseini F. (2023). Mechanisms of immune evasion of monkeypox virus. Front. Microbiol..

[84] Fang D., Liu Y., Dou D., Su B. (2024). The unique immune evasion mechanisms of the mpox virus and their implication for developing new vaccines and immunotherapies. Virol. Sin..

[85] Dashraath P., Nielsen-Saines K., Mattar C., Musso D., Tambyah P., Baud D. (2022). Guidelines for pregnant individuals with monkeypox virus exposure. Lancet.

[86] Khalil A., Samara A., O’Brien P., Ladhani S. (2022). Call for a unified approach to Monkeypox infection in pregnancy: Lessons from the COVID-19 pandemic. Nat. Commun..

[87] Brown K., Leggat P.A. (2016). Human Monkeypox: Current State of Knowledge and Implications for the Future. Trop. Med. Infect. Dis..

[88] Rizk J.G., Lippi G., Henry B.M., Forthal D.N., Rizk Y. (2022). Prevention and Treatment of Monkeypox. Drugs.

[89] Almehmadi M., Allahyani M., Alsaiari A.A., Alshammari M.K., Alharbi A.S., Hussain K.H., Alsubaihi L.I., Kamal M., Alotaibi S.S., Alotaibi A.N. (2022). A Glance at the Development and Patent Literature of Tecovirimat: The First-in-Class Therapy for Emerging Monkeypox Outbreak. Viruses.

[90] Drosu N.C., Edelman E.R., Housman D.E. (2020). Tenofovir prodrugs potently inhibit Epstein-Barr virus lytic DNA replication by targeting the viral DNA polymerase. Proc. Natl. Acad. Sci. USA.

[91] Sherwat A., Brooks J.T., Birnkrant D., Kim P. (2022). Tecovirimat and the Treatment of Monkeypox—Past, Present, and Future Considerations. N. Engl. J. Med..

[92] Warner B.M., Klassen L., Sloan A., Deschambault Y., Soule G., Banadyga L., Cao J., Strong J.E., Kobasa D., Safronetz D. (2022). In vitro and in vivo efficacy of tecovirimat against a recently emerged 2022 monkeypox virus isolate. Sci. Transl. Med..

[93] Lozano J.M., Muller S. (2023). Monkeypox: Potential vaccine development strategies. Trends Pharmacol. Sci..

[94] Eggers M., Exner M., Gebel J., Ilschner C., Rabenau H.F., Schwebke I. (2022). Hygiene and disinfection measures for monkeypox virus infections. GMS Hyg. Infect. Control..

[95] Goyal L., Ajmera K., Pandit R., Pandit T. (2022). Prevention and Treatment of Monkeypox: A Step-by-Step Guide for Healthcare Professionals and General Population. Cureus.

[96] Reynolds M.G., Damon I.K. (2012). Outbreaks of human monkeypox after cessation of smallpox vaccination. Trends Microbiol..

[97] Poland G.A., Kennedy R.B., Tosh P.K. (2022). Prevention of monkeypox with vaccines: A rapid review. Lancet Infect. Dis..

[98] Townsend M.B., Keckler M.S., Patel N., Davies D.H., Felgner P., Damon I.K., Karem K.L. (2013). Humoral immunity to smallpox vaccines and monkeypox virus challenge: Proteomic assessment and clinical correlations. J. Virol..

[99] Mason L.M.K., Betancur E., Riera-Montes M., Lienert F., Scheele S. (2024). MVA-BN vaccine effectiveness: A systematic review of real-world evidence in outbreak settings. Vaccine.

[100] Grabenstein J.D., Hacker A. (2024). Vaccines against mpox: MVA-BN and LC16m8. Expert Rev. Vaccines.

[101] Saadh M.J., Ghadimkhani T., Soltani N., Abbassioun A., Daniel Cosme Pecho R., Taha A., Jwad Kazem T., Yasamineh S., Gholizadeh O. (2023). Progress and prospects on vaccine development against monkeypox infection. Microb. Pathog..

[102] Mayer L., Weskamm L.M., Addo M.M. (2024). Next-generation mpox vaccines: Efficacy of mRNA-1769 compared to modified vaccinia virus Ankara in non-human primates. Signal Transduct. Target. Ther..

[103] Eto A., Saito T., Yokote H., Kurane I., Kanatani Y. (2015). Recent advances in the study of live attenuated cell-cultured smallpox vaccine LC16m8. Vaccine.

[104] Greenberg R.N., Kennedy J.S. (2008). ACAM2000: A newly licensed cell culture-based live vaccinia smallpox vaccine. Expert Opin. Investig. Drugs.

[105] Kenner J., Cameron F., Empig C., Jobes D.V., Gurwith M. (2006). LC16m8: An attenuated smallpox vaccine. Vaccine.

[106] Tang H., Zhang A. (2023). Human mpox: Biology, epidemiology, therapeutic options, and development of small molecule inhibitors. Med. Res. Rev..

[107] Khamees A., Awadi S., Al-Shami K., Alkhoun H.A., Al-Eitan S.F., Alsheikh A.M., Saeed A., Al-Zoubi R.M., Zoubi M.S.A. (2023). Human monkeypox virus in the shadow of the COVID-19 pandemic. J. Infect. Public Health.

[108] National Institutes of Health (2022). Clinical Trial Evaluating Monkeypox Vaccine Begins NIH Trial is Evaluating Intradermal Delivery to Expand the Vaccine Supply.

[109] Ghazy R.M., Elrewany E., Gebreal A., ElMakhzangy R., Fadl N., Elbanna E.H., Tolba M.M., Hammad E.M., Youssef N., Abosheaishaa H. (2023). Systematic Review on the Efficacy, Effectiveness, Safety, and Immunogenicity of Monkeypox Vaccine. Vaccines.

[110] Beaney A. (2024). Moderna mpox mRNA vaccine shows early promise in monkey study. Clinical Trials Arena.

[111] O’Laughlin K., Tobolowsky F.A., Elmor R., Overton R., O’Connor S.M., Damon I.K., Petersen B.W., Rao A.K., Chat-ham-Stephens K., Yu P. (2022). Clinical Use of Tecovirimat (Tpoxx) for Treatment of Monkeypox Under an Investigational New Drug Protocol—United States, May–August 2022. MMWR Morb. Mortal. Wkly. Rep..

[112] National Institutes of Health (2024). The Antiviral Tecovirimat is Safe but Did Not Improve Clade I Mpox Resolution in Democratic Republic of the Congo.

[113] National Institutes of Health (2024). NIH Study Finds Tecovirimat Was Safe but Did Not Improve Mpox Resolution or Pain.

[114] Reynolds M.G., Carroll D.S., Karem K.L. (2012). Factors affecting the likelihood of monkeypox’s emergence and spread in the post-smallpox era. Curr. Opin. Virol..

